# Effects of Manganese Hydroxychloride on Growth Performance, Antioxidant Capacity, Tibia Parameters and Manganese Deposition of Broilers

**DOI:** 10.3390/ani11123470

**Published:** 2021-12-06

**Authors:** Yongbo Sun, Shixia Geng, Tianyao Yuan, Ying Liu, Yuxin Zhang, Yuting Di, Juntao Li, Liying Zhang

**Affiliations:** State Key Laboratory of Animal Nutrition, Ministry of Agriculture and Rural Affairs Feed Industry Centre, China Agricultural University, Beijing 100193, China; ybsun2014@163.com (Y.S.); gengshixia@163.com (S.G.); tyyuan2018@163.com (T.Y.); liuyingcau2021@163.com (Y.L.); s20203040632@cau.edu.cn (Y.Z.); dyt971004@163.com (Y.D.); wacau@163.com (J.L.)

**Keywords:** Mn hydroxychloride, broiler, antioxidant capacity, tibia parameters, Mn deposition

## Abstract

**Simple Summary:**

Manganese is a vital trace element for the growth of broilers. In order to meet the requirement of manganese in broiler production, the additives of manganese sources are usually added into the diet for broilers. Manganese hydroxychloride is a category of hydroxy trace minerals. The present study investigated the effect of dietary supplemental manganese as manganese hydroxychloride for growth performance, antioxidant capacity, tibial quality, and manganese deposition of broilers and recommended that optimal supplementation with manganese as manganese hydroxychloride in diets for broilers was 50–90 mg/kg. This study provides a rational recommendation for the application of manganese hydroxychloride in broiler diets.

**Abstract:**

This study was conducted to investigate the effects of dietary supplementation with manganese hydroxychloride (MHC) on production performance, antioxidant capacity, tibial quality, and manganese (Mn) deposition of broilers. A total of 756 one-day-old male Arbor Acres broilers were randomly allotted to 7 treatments of 6 replicates with 18 broilers per replicate. Broilers were fed corn-soybean meal basal diets supplemented of 100 mg/kg Mn as Mn sulfate (MnSO_4_), or 0, 20, 40, 60, 80, 100 mg/kg Mn as MHC for 42 days. The growth performance of broilers was not affected by dietary MnSO_4_ or MHC (*p* > 0.05), whereas the dressing percentage increased linearly (*p* < 0.05) with increasing of dietary MHC addition level. The activities of catalase (CAT) and manganese superoxide dismutase (MnSOD), and total antioxidant capability (T-AOC) in serum and liver on day 42 increased linearly (*p* < 0.05) with increasing of dietary MHC level, while malondialdehyde (MDA) concentration reduced linearly (*p* < 0.05). The length, strength, and density index of tibia increased linearly (*p* < 0.05) on day 21 as MHC supplementation level increased; there were no differences between MnSO_4_ group and 40–100 mg/kg Mn as MHC groups in tibial parameters of broilers (*p* > 0.05). As supplemental MHC levels increased, the Mn contents in heart, liver, kidney, and tibia increased linearly on day 42 (*p* < 0.05). In summary, dietary supplementation with MHC improved antioxidant capacity, bone quality, and Mn contents in broilers, but no effects on growth performance were detected. Based on the results of this study, dietary inclusion of 50–90 mg/kg Mn in the form of MHC to broilers is recommended.

## 1. Introduction

As an essential trace element, manganese (Mn) is a component or activator of many essential enzymes, such as arginase, pyruvate carboxylase, hydrolases, etc., which are involved in carbohydrate, lipid, and protein metabolism and many crucial biochemical reactions [1,2]. It is also an essential cofactor of chondroitin sulfate synthesis that is closely related to the bone formation of broilers [3]. Otherwise, Mn plays a vital role in antioxidant and immune systems of animals [4,5,6]. As recommended by the National Research Council (NRC), the content of Mn in the diet for broilers should be at least 60 mg/kg [7]. However, the content of Mn is just around 30 mg/kg in the diet mainly consisting of corn and soybean meal and its utilization rate of Mn is very low [8]. In order to meet the requirement of Mn in broiler production, the additives of Mn sources are usually added into the diet for broilers.

At present, additives of Mn source which is usually used in broiler diets include inorganic Mn (such as Mn sulfate, Mn carbonate, and Mn oxide) and organic Mn (such as amino acid-chelated Mn and protein Mn). For traditional inorganic sources of Mn, they are easy to deliquescence and the utilization rate is relatively low, although they are cheaper [9,10]. Organic sources of Mn have shown excellent chemical stability and high absorption efficiency, but they have not been widely applied in production because of their uneven product quality, inconsistent effect, and high cost [9,11,12]. Thus, it is very important to develop new sources of Mn with better efficacy and relatively low cost.

Manganese hydroxychloride (MHC), also known as basic Mn chloride or tribasic Mn chloride, is a category of hydroxy trace minerals. The solubility of MHC in water is minimal, but it becomes more soluble under acidic conditions in intestine [13]. MHC contains strong covalent bonds that are similar to that of organic minerals and have a special crystalline structure, which may be beneficial for the stability in the diet and better absorption in the intestine. MHC has been approved as a feed additive by the European Food Safety Authority (EFSA) [14]. Previous studies showed that dietary supplementation with MHC could increase egg yolk and shell Mn levels of laying hens, and improve feed conversion of broilers [15,16]. A study conducted with pigs also showed that dietary inclusion of MHC improved the growth rate and feed intake compared with that of MnSO_4_ [17]. However, there is limited literature about the suitable addition level of MHC for broilers, and the relative effect compared with typical inorganic Mn has not been reported. Therefore, the main aim of this study was to explore the suitable level of Mn as MHC in the broiler diet by investigating the effects of dietary MHC supplementation on growth performance, slaughter traits, antioxidant capacity, tibial quality, and tissue Mn level of broilers.

## 2. Materials and Methods

### 2.1. Experimental Material

Manganese hydroxychloride (MHC) was supplied by Changsha Xingjia Bio-Engineering Co., Ltd. (Changsha, China). The molecular formula of MHC is Mn_2_(OH)_3_Cl and the analyzed content of Mn was 55.64%. The feed grade MnSO_4_ was purchased from Beijing Tongli Xingke Agricultural Technology Co., Ltd. (Beijing, China), the analyzed Mn concentration was 31.94%.

### 2.2. Animals, Experimental Design and Management

This study was performed on the Fengning Research Unit of China Agricultural University (Chengde, Hebei, China). A total of 756 one-day-old Arbor Acres male broiler chicks with an average body weight of 44.28 ± 1.74 g were obtained from Arbor Acres Poultry Breeding Company (Beijing, China). All birds were weighed and randomly assigned to 7 treatments with 6 replicates and 18 chicks in each replicate. The diets consisted of a corn and soybean diet with 0 (control group), 20, 40, 60, 80, or 100 mg /kg Mn as MHC, and 100 mg/kg Mn as MnSO_4_ (positive control), respectively. The recommended Mn content for broilers is 120 mg/kg during 1–3 weeks and 100 mg/kg during 4–6 weeks by the Feeding Standard of Chicken in China. Additionally, we searched recent related articles on broiler experiments in *Animals* and also found that the amount of added Mn in the diet was around 100 mg/kg [18,19,20,21,22]. So we added 100 mg/kg Mn in the form of MnSO_4_ as the positive control. This experiment lasted for 42 d, and all broilers were fed with starter diets from day 0 to day 21, and then fed with grower diets until the end of the trial. All diets were fed in mash form. Except for Mn, the corn-soybean meal basal diet was formulated according to the recommendations of the nutritional requirements of broilers (NRC, 1994) [7]. The ingredients compositions and nutritional levels of basal diets are shown in Table 1.

The experiment was performed on the Fengning Research Base of China Agricultural University (Chengde, Hebei, China). All broilers were raised in 3-layer cages (0.093 m^2^ per bird) with six birds per cage in an environmentally controlled room. Feed and water were offered ad libitum throughout the experiment. The lighting program was 23 h light: 1 h dark per day. The room temperature was maintained at 35 °C for the first 3 day and gradually reduced by 3 °C each week until it reached to final temperature of 24 °C. All chicks were inoculated with Newcastle disease vaccine on day 7 and day 21, and infectious bursal disease vaccine on day 14 and day 28.

### 2.3. Growth Performance

Body weight and feed intake per replicate were recorded on days 21 and 42 of the experiment. Average daily gain (ADG), average daily feed intake (ADFI), and feed to gain ratio (F:G) were calculated from days 0 to 21, 22 to 42, and 0 to 42.

### 2.4. Sample Collection

On day 21 and 42 after fasting for 12 h, 6 broilers approximating the average weight from each treatment (one bird per replicate) were selected to collect blood samples from the wing vein of broilers. Blood samples were allowed to stand for 30 min at room temperature, followed by centrifugation at 3600× *g* for 15 min. Then, serum samples were collected and stored at −20 °C for further analysis. The selected broilers were euthanized by jugular vein bleeding after stunning using 60% concentration of CO_2_ gas. The tibia, liver, heart, kidney, and breast muscle samples were collected and stored at −20 °C for subsequent analysis. In addition, live weight, carcass weight, eviscerated weight, breast weight, thigh muscle weight, and abdominal fat weight of broilers were measured on day 42. Dressing percentage and eviscerated percentage were expressed as a percentage of its initial live weight, while breast meat percentage, leg meat percentage, and abdominal fat percentage were expressed as a percentage of the eviscerated weight.

### 2.5. Sample Analysis

#### 2.5.1. Nutrients Level of Diets

Diets were analyzed according to the methods of the Association of Official Analytical Chemists (AOAC, 2000) for total phosphorus (method 995.11), calcium (method 927.02), crude protein (method 988.05), and amino acids (method 994.12). For dietary methionine determination, performic acid oxidation was performed prior to acid hydrolysis with 6 M HCl. The Mn concentration of MHC, MnSO_4_, and diets was determined by Z-2000 flame atomic absorption spectrometry (Hitachi, Tokyo, Japan).

#### 2.5.2. Antioxidant Capacity

Total antioxidant capacity (T-AOC), Mn containing superoxide dismutase (MnSOD) and catalase (CAT) activities, and malondialdehyde (MDA) contents in serum and liver of broilers were measured with commercial kits (Sino-UK Institute of Biological Technology, Beijing, China) according to the manufacturer’s protocol.

#### 2.5.3. Tibia Indicator

After taking the left tibia, muscles, cartilage, and membranes of it were removed. The length and diameter of tibia were measured using a vernier caliper and then weighed. The density index of the tibia was measured using a dual-energy X-ray absorptiometry bone densitometer (Hologic, Bedford, MA, USA), and the breaking strength was measured using a TA.XT plus texture analyser (Stable Microsystems, Surrey, UK).

#### 2.5.4. Manganese Contents

The contents of Mn in heart, liver, kidney, tibia, and breast muscles samples of broilers on day 42 were determined by inductively coupled plasma mass spectrometry (Agilent 7500, Agilent Technologies, Tokyo, Japan) after microwave digestions with nitric acid.

### 2.6. Statistical Analysis

All data were subjected to one-way ANOVA using the general linear model (GLM) procedure of SAS 9.2 (SAS Institute Inc., Cary, NC, USA). Differences among treatments were further compared using Duncan’s multiple range test. Orthogonal polynomial contrasts were used to analyze the linear and quadratic responses to MHC levels. Also, a quadratic regression fitting curve model [*y* = a*x*^2^ + b*x* + c, the best addition level *x* = −b/(2a)] was performed using GraphPad Prism 7 (GraphPad Software Inc.; San Diego, CA, USA) to evaluate the optimal MHC addition levels. A *p*-value of less than 0.05 was considered to be statistically significant.

## 3. Results

### 3.1. Mn Contents in Experimental Diets

The analyzed Mn content in the starter and grower diets are presented in Table 2. The analyzed Mn concentrations in diets supplemented with 0, 20, 40, 60, 80, or 100 mg /kg Mn as MHC, and 100 mg/kg Mn as MnSO_4_ were 34.4, 53.3, 73.1, 91.7, 110.5, 139.9, and 150.4 mg/kg for the starter diets, and 37.0, 60.8, 77.1, 99.6, 119.0, 142.5 and 151.1 mg/kg for the grower diets, respectively.

### 3.2. Growth Performance

The effects of dietary supplementation with MHC on the growth performance of broilers are presented in Table 3. There were no significant differences in ADG, ADFI, and F:G among all treatments were detected during days 0–21, 22–42, and 0–42 (*p* > 0.05).

### 3.3. Carcass Characteristics

As shown in Table 4, the dressing percentage of broilers increased linearly (*p* < 0.05) on day 42 with the increase of dietary MHC addition level. The percentage of breast muscle, leg muscle, and abdominal fat had not been significantly affected by dietary MHC or MnSO_4_ (*p* > 0.05).

### 3.4. Antioxidant Capacity

The effects of dietary supplementation with MHC on the antioxidant capacity in serum of broiler are shown in Table 5. Dietary supplementation with MHC linearly increased the activities of CAT and MnSOD in serum on day 21 (*p* < 0.01), and the activities of serum CAT and MnSOD and T-AOC also increased (linear, *p* < 0.05; quadratic, *p* < 0.05) as supplemental MHC level increased on day 42 (*p* < 0.05), whereas MDA level in serum decreased linearly on day 21 and 42 (*p* < 0.01). No differences were observed in these parameters among MnSO_4_ and 40–60 mg/kg MHC groups on day 42 (*p* > 0.05).

As shown in Table 6, diet supplemented with MnSO_4_ or 80–100 mg/kg Mn as MHC increased MnSOD activity of liver on day 21 (*p* < 0.05) compared to that of control group. With the increasing of dietary MHC supplementation level, the activities of MnSOD (linear, *p* < 0.05; quadratic, *p* < 0.05) and CAT (linear, *p* < 0.05), and T-AOC (linear, *p* < 0.05; quadratic, *p* < 0.05) of liver increased on day 42, while MDA level decreased (linear, *p* < 0.05). Additionally, CAT activity and T-AOC with 60 mg/kg MHC treatment were greater than those in the MnSO_4_ treatment (*p* < 0.05).

### 3.5. Tibial Parameters

Effects of dietary supplementation with MHC on tibial parameters of broilers are presented in Table 7. With the increase of MHC supplementation level, the length, strength, and density index of tibia increased linearly on day 21 (*p* < 0.05). However, there were no significant differences in all measured tibial parameters among treatments were detected on day 42 (*p* > 0.05). Additionally, no differences in tibial parameters between MnSO_4_ group and 40–100 mg/kg MHC groups were observed (*p* > 0.05).

### 3.6. Mn Contents in Tissues

As shown in Table 8, the contents of Mn in heart, liver, kidney, and tibia of broilers increased linearly on day 42 (*p* < 0.05) as supplemental MHC level increased. However, there was no difference in Mn level of serum and chicken breast muscle among all groups were detected (*p* > 0.05). Additionally, no differences in the Mn contents in liver and tibia of broilers between MnSO_4_ and 80 mg/kg MHC group were observed (*p* > 0.05).

### 3.7. The Optimal Supplementation Level

**Figure 1:**
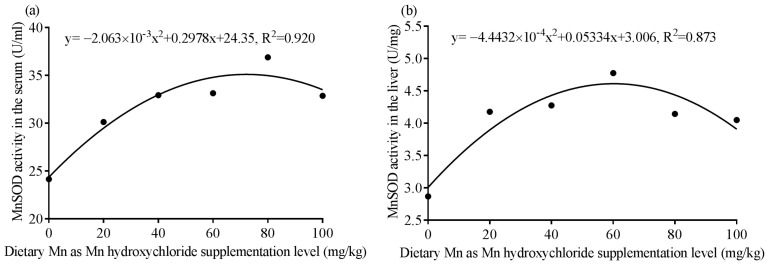
The quadratic fit model of dietary manganese (Mn) supplementation level as Mn hydroxychloride (MHC) and Mn superoxide dismutase (MnSOD) activities in the serum (**a**) and liver (**b**) of broilers on day 42.

According to the quadratic regression curve of MnSOD activities to dietary MHC level in serum and liver on day 42 (Figure 1), the optimal dietary supplementation level of Mn in the form of MHC is 72.18 mg/kg and 60.18 mg/kg, respectively; and the suitable inclusion level is 53.77–90.58 mg/kg and 49.88–70.47 mg/kg, respectively.

For MnSOD activity in the serum, the quadratic model was *y* = −2.063 × 10^−3^*x*^2^ + 0.2978*x* + 24.35, R^2^ = 0.920, *p* = 0.023, the best addition level of Mn as MHC is 72.18 mg/kg, and the optimal addition range is 53.77–90.58 mg/kg. For MnSOD activity in the liver, the quadratic model was *y* = −4.4432 × 10^−4^*x*^2^ + 0.05334*x* + 3.006, R^2^ = 0.873, *p* = 0.046, the best addition level of Mn as MHC is 60.18 mg/kg, and the optimal addition range is 49.88–70.47mg/kg.

## 4. Discussion

The reports related to the effects of Mn on growth performance of broilers were inconsistent. Many studies have shown that dietary supplementation with different chemical forms of Mn such as Mn propionate [12], Mn proteinates [23], Mn oxide [24], Mn sulfate [25], Mn fumarate [26], or Mn amino acid chelate [27] did not significantly affect ADG, ADFI, and F:G of broilers. However, Meng et al. [28] found dietary inclusion of 50 mg/kg Mn as Mn methionine hydroxyl analog chelated could improve ADG and ADFI of broilers. Otherwise, Ognik et al. [29] reported that diet supplemented with 50 or 100 mg/kg Mn in the form of Mn oxide nanoparticles decreased F:G of turkeys. At present, there are a few studies on the effect of MHC on broiler, and the results are inconsistent. Conly et al. [30] showed that diet (45 mg/kg Mn) supplemented with 30–130 mg/kg Mn in the form of MHC had no significant effect on feed intake, body weight, and F:G of Cobb 500 broilers. The present study also showed that dietary (37 mg/kg Mn) supplementation of 20–100 mg/kg Mn as MHC had no significant effect on the ADG, ADFI, and F:G of AA broilers. However, Jasek et al. [16] reported dietary (40 mg/kg Mn) inclusion of 40–160 mg/kg Mn as MHC decreased F:G of Ross 708 broilers. These disparities in results among studies may be due to the difference in experiment broiler breed, Mn content in basal diet, source and addition level of Mn.

Carcass characteristics are important parameters for evaluating the meat production performance of broilers. Studies have shown that dietary supplementation of 100 mg/kg Mn as MnO and Mn_2_O_3_ nanoparticles improved the carcass yield of turkeys [31], and supplementation of 100 mg/kg Mn in the form of MnSO_4_ or amino acid chelated Mn reduced the abdominal fat rate of broilers [25,32,33]. The present study showed that dietary inclusion of MHC did not significantly affect slaughter characteristics of broilers, and there were no differences with MnSO_4_ were detected. Matuszewski et al. [34] also reported that dietary supplementation with Mn_2_O_3_ and Mn_2_O_3_ nanoparticles (21–70 mg/kg) did not significantly affect any slaughter characteristics of broilers. Further experiments need to be conducted with several Mn sources and levels under the same condition, especially in large-scale commercial farm conditions to confirm the results.

Parameter CAT, T-AOC, and MDA are usually used to evaluate the antioxidant ability of animals. As a component of MnSOD, Mn can improve broiler’s antioxidant ability by catalyzing the reduction of superoxide anion to hydrogen peroxide [35]. An in vitro study indicated that MnSOD activity and mRNA expression level in chick embryonic myocardial cells were improved by 1.0 mmol/L of Mn as MnCl_2_ treatment [36]. Studies also have shown that dietary inclusion of Mn as MnSO_4_, Mn methionine, or Mn oxide improved MnSOD, CAT, and GSH-Px activities and T-AOC, while reducing MDA level in serum, liver, and leg muscle of broilers [25,37]. In this study, dietary inclusion of MHC increased MnSOD and CAT activities and T-AOC, while decreasing MDA content in serum and liver of broilers. Therefore, dietary supplementation with MHC can improve the antioxidant capacity and reduce oxidative damage of broilers by improving antioxidative enzyme activities and reducing peroxidation products content. The increase of MnSOD activity may be due to that Mn activating protein kinase C and protein tyrosine kinase [35], altering specificity protein 1 and activating protein-2 DNA-binding activities, and enhancing MnSOD binding protein RNA-binding activity at the translational level [38]. The increase of other antioxidant enzymes may be related to the activation of Nrf2 signaling pathway by Mn treatment [39]. Additionally, due to the MnSOD activities in serum and liver showing significant quadratic response to dietary MHC addition level, it can be also concluded that the optimal dietary supplementation level of Mn in the form of MHC is 50–90 mg/kg according to the quadratic regression curve of MnSOD activities in the serum and liver. The broken-line models are not shown here because the data better fitted the quadratic model (R^2^ = 0.920 and 0.873 for the serum and liver, respectively) than a broken line (R^2^ = 0.820 and 0.844 for the serum and liver, respectively).

Studies showed that Mn deficiency experimental model of broilers was successfully established at the dose of 40 mg/kg which can affect the normal development of tibia by inhibiting the vitality of osteoblasts and chondrocyte proliferation and promoting chondrocyte apoptosis in the tibia [40,41], disordering the level of bone regulatory hormones and enzymes of bone metabolism in the serum [42], and leading to metaphyseal osteoporosis [40]. The length, weight, diameter, breaking strength, or density index of tibia are usual parameters to be used for evaluating development of tibia. Studies reported that dietary supplementation of Mn as MnSO_4_, MnCO_3_, and MnO could increase the length, weight, diameter, breaking strength, and density index of tibia in broilers [42,43,44], reduce the incidence of leg abnormalities [25,32]. However, Bozkurt et al. [37] reported that dietary supplementation of Mn as Mn-methionine and MnO with levels 12.5, 25, and 50 mg/kg has no effect on the weight, length, diameter, and density index of tibia in broilers. It is assumed that the different bioavailability of different chemical forms in Mn sources may be the reason which resulted in these inconsistent results. In this study, it is shown that dietary supplementation of Mn as MHC increased length and density index of tibia in the early growth stage of broilers, which may be due to that broilers had low feed intake, and rapid bone growth and development, especially during the first two week of post-hatch age when the bone is not completely formed [45]; whereas, broilers can obtain sufficient Mn for bone growth due to the increase of feed intake at the late growth stage.

The source and addition level of Mn in diet may directly affect the Mn content of broiler tissue. Dietary supplementation of Mn in the form of MnSO_4_, MnO, or Mn fumarate could improve Mn levels in tibia, liver, and kidney of broilers [26,46]. In the present study, dietary supplementation with MHC improved Mn levels in the heart, liver, kidney, and tibia, which was agreed with the study conducted by Conly et al. [30]. However, it is also found that dietary Mn level has no significant effect on the Mn content in serum and breast muscle. European Food Safety Authority (2016) also reported that dietary MHC or MnSO_4_ increased Mn levels in the liver and tibia but did not significantly affect Mn level in breast muscle [14]. This may be due to the weak ability to deposit Mn in breast muscle, where mitochondria are not abundant. And most of Mn in serum was transferred to other organizations.

According to the results of the present study, it seems that the efficacy of MHC is a little higher than that of MnSO_4_ on the basis of some measured indicators including antioxidant capacity, tibial parameters, and Mn contents in liver and tibia. So it is assumed that MHC maybe have higher relative bioavailability than that of MnSO_4_. MHC was combined by covalent bonds between Mn, hydroxy groups as well as chloride ions, creating a stronger chemical bond than traditional sulfate minerals [14]. The covalent bonds possessed by hydroxychloride minerals can also reduce the reaction of minerals with other components in feeds [47], so its bioavailability could be potentially improved. However, the accurate relative biological availability of Mn as MHC to Mn sulfate has not been reported, which needs to be further studied.

## 5. Conclusions

Dietary inclusion of MHC can improve the antioxidant capacity, bone quality, and Mn deposition of broilers, but no effects on growth performance were detected. Dietary inclusion of 50–90 mg/kg Mn as MHC is recommended in broilers.

## Figures and Tables

**Table 1:** Ingredient composition and nutrient levels of the basal diets (%, as-fed basis).

Item	Starter Diets (0~21 Days of Age)	Grower Diets (22~42 Days of Age)
Ingredient		
Corn	60.13	61.53
Soybean meal	32.50	31.70
Fish meal	2.00	0.00
Soybean oil	1.50	3.00
Dicalcium phosphate	1.50	1.70
Limestone	1.34	1.15
DL-Methionine (98%)	0.23	0.12
NaCl	0.30	0.30
Premix ^1^	0.50	0.50
Total	100.00	100.00
Nutrient composition ^2^		
Metabolizable energy (MJ/kg)	12.59	13.22
Crude protein	21.75	20.27
Calcium	0.98	0.88
Total phosphorus	0.68	0.63
Total lysine	1.10	0.95
Total methionine + cysteine	0.85	0.73
Mn (mg/kg)	34.4	37.0

^1^ Premix provided per kg of diet: vitamin A, 9000 IU; vitamin D3, 3000 IU; vitamin E, 24 mg; vitamin K3, 1.8 mg; vitamin B1, 2.0 mg; riboflavin, 5 mg; vitamin B6, 3.0 mg; vitamin B12, 0.1 mg; nicotinic acid, 40 mg; calcium pantothenate, 15 mg; folic acid, 1 mg; biotin, 0.05 mg; choline chloride, 500 mg; Fe, 80 mg; Cu, 20 mg; Zn, 90 mg; iodine, 0.35 mg; Se, 0.3 mg. ^2^ Metabolizable energy was calculated values using NRC (1994) values; others were analyzed values.

**Table 2:** Analyzed manganese (Mn) contents in experimental diets.

Item	Added Mn, mg/kg	Analyzed Mn Contents, mg/kg	
		Day 0 to 21	Day 22 to 42
Mn as MnSO_4_	100	150.4	151.1
Mn as manganese hydroxychloride	0	34.4	37.0
	20	53.3	60.8
	40	73.1	77.1
	60	91.7	99.6
	80	110.5	119.0
	100	139.9	142.5

**Table 3:** Effect of dietary supplementation with manganese hydroxychloride on growth performance of broilers ^1^.

Item ^2^	Mn as MnSO_4_ (mg/kg)	Mn as Manganese Hydroxychloride (mg/kg)						SEM	*p*-Value		
	100	0	20	40	60	80	100		ANOVA	Linear	Quadratic
Day 0~21											
ADG (g)	32.60	31.96	33.01	32.37	33.37	32.05	33.25	0.478	0.241	0.235	0.638
ADFI (g)	43.97	43.27	44.18	43.66	44.52	43.42	44.33	0.575	0.674	0.396	0.644
F:G	1.35	1.36	1.34	1.35	1.34	1.35	1.33	0.011	0.650	0.374	0.857
Day 22~42											
ADG (g)	71.58	68.86	69.83	69.46	72.01	71.71	70.48	1.357	0.559	0.145	0.373
ADFI (g)	136.48	134.34	135.92	134.45	138.72	136.03	135.71	1.596	0.549	0.380	0.314
F:G	1.91	1.95	1.95	1.94	1.93	1.90	1.93	0.028	0.800	0.245	0.748
Day 0~42											
ADG (g)	52.09	50.41	51.42	50.91	52.69	51.88	51.86	0.835	0.552	0.128	0.389
ADFI (g)	90.23	88.81	90.05	89.06	91.62	89.73	90.02	0.941	0.475	0.299	0.301
F:G	1.73	1.76	1.75	1.75	1.74	1.73	1.74	0.019	0.891	0.227	0.780

^1^ Values are expressed as means of six replicates of 18 birds per pen. ^2^ ADG, average daily gain; ADFI, average daily feed intake; F:G, feed: gain ratio.

**Table 4:** Effect of dietary supplementation with manganese hydroxychloride on slaughter performance of broilers at the age of day 42 (%) ^1^.

Item	Mn as MnSO_4_ (mg/kg)	Mn as Manganese Hydroxychloride (mg/kg)						SEM	*p*-Value		
	100	0	20	40	60	80	100		ANOVA	Linear	Quadratic
Carcass weight/body weight	92.24	91.09	91.57	91.18	91.74	91.60	92.54	0.454	0.296	0.048	0.442
Eviscerated weight/body weight	69.72	69.04	70.21	70.14	71.45	70.82	70.58	0.639	0.101	0.057	0.130
Breast weight/eviscerated weight	25.04	25.76	25.42	25.08	25.90	24.27	25.98	0.622	0.473	0.777	0.382
Leg weight/eviscerated weight	22.31	22.21	21.74	21.35	21.65	21.73	22.26	0.632	0.927	0.921	0.261
Abdominal fat weight/eviscerated weight	1.34	1.53	1.43	1.31	1.40	1.38	1.42	0.143	0.925	0.589	0.376

^1^ Values represent the means of six pens (*n* = 6) per treatment.

**Table 5:** Effect of dietary supplementation with manganese hydroxychloride on antioxidant capacity in serum of broilers ^1^.

Item ^2^	Mn as MnSO_4_ (mg/kg)	Mn as Manganese Hydroxychloride (mg/kg)						SEM	*p*-Value		
	100	0	20	40	60	80	100		ANOVA	Linear	Quadratic
Day 21											
CAT (U/mL)	67.21 ^a^	48.37 ^b^	61.12 ^a^	64.13 ^a^	64.33 ^a^	64.12 ^a^	64.52 ^a^	2.823	0.001	<0.001	0.052
MnSOD (U/mL)	54.19 ^a^	29.23 ^b^	34.79 ^b^	39.87 ^b^	40.29 ^b^	56.05 ^a^	57.44 ^a^	4.589	<0.001	<0.001	0.610
MDA (nmol/mL)	4.24	5.26	5.22	4.63	4.49	4.22	4.08	0.299	0.055	0.002	0.782
T-AOC (U/mL)	11.04	10.75	10.60	11.03	10.67	10.72	10.69	0.217	0.369	0.894	0.666
Day 42											
CAT (U/mL)	66.65 ^b^	52.89 ^c^	66.08 ^b^	66.98 ^b^	67.29 ^b^	78.28 ^a^	75.89 ^a^	1.755	<0.001	<0.001	0.027
MnSOD (U/mL)	25.59 ^b^	24.14 ^c^	30.12 ^b^	32.93 ^ab^	33.13 ^ab^	36.88 ^a^	32.86 ^ab^	1.431	<0.001	<0.001	0.002
MDA (nmol/mL)	4.11 ^c^	5.75 ^a^	5.16 ^b^	4.67 ^bc^	4.78 ^bc^	4.60 ^bc^	4.15 ^c^	0.196	<0.001	<0.001	0.233
T-AOC (U/mL)	11.68 ^b^	8.71 ^d^	10.09 ^c^	11.11 ^b^	11.12 ^b^	12.92 ^a^	12.02 ^ab^	0.275	<0.001	<0.001	0.012

^1^ Values represent the means of six pens (*n* = 6) per treatment. ^2^ CAT, Catalase; MnSOD, Manganese superoxide dismutase; MDA, Malonaldehyde; T-AOC, Total antioxidant capacity. ^a, b, c^ Means within a row with different superscripts are significantly different at the *p*-value indicated for ANOVA.

**Table 6:** Effect of dietary supplementation with manganese hydroxychloride on antioxidant capacity in liver of broilers ^1^.

Item ^2^	Mn as MnSO_4_ (mg/kg)	Mn as Manganese Hydroxychloride (mg/kg)						SEM	*p*-Value		
	100	0	20	40	60	80	100		ANOVA	Linear	Quadratic
Day 21											
CAT (U/mg protein)	4.17	3.02	3.30	3.45	3.26	3.59	3.93	0.332	0.220	0.057	0.723
MnSOD (U/mg protein)	4.94 ^ab^	2.68 ^c^	3.38 ^bc^	4.05 ^abc^	4.33 ^abc^	4.46 ^ab^	5.58 ^a^	0.537	0.013	<0.001	0.995
MDA (nmol/mg protein)	0.36	0.49	0.49	0.47	0.44	0.48	0.40	0.037	0.145	0.097	0.675
T-AOC (U/mg protein)	1.46	1.39	1.46	1.44	1.48	1.25	1.39	0.075	0.355	0.313	0.432
Day 42											
CAT (U/mg protein)	4.48 ^bc^	3.96 ^c^	4.50 ^bc^	4.56 ^bc^	5.22 ^a^	4.74 ^ab^	4.66 ^ab^	0.199	0.008	0.007	0.084
MnSOD (U/mg protein)	4.11 ^a^	2.87 ^b^	4.48 ^a^	4.27 ^a^	4.77 ^a^	4.14 ^a^	4.05 ^a^	0.290	0.004	0.021	0.002
MDA (nmol/mg protein)	0.47 ^ab^	0.55 ^a^	0.49 ^ab^	0.48 ^ab^	0.42 ^b^	0.41 ^b^	0.44 ^b^	0.028	0.018	0.001	0.103
T-AOC (U/mg protein)	1.24 ^b^	1.07 ^c^	1.54 ^ab^	1.64 ^a^	1.73 ^a^	1.61 ^a^	1.53 ^ab^	0.113	0.002	0.015	0.003

^1^ Values represent the means of six pens (*n* = 6) per treatment. ^2^ CAT, Catalase; MnSOD, Manganese superoxide dismutase; MDA, Malonaldehyde; T-AOC, Total antioxidant capacity. ^a, b, c^ Means within a row with different superscripts are significantly different at the *p*-value indicated for ANOVA.

**Table 7:** Effect of dietary supplementation with manganese hydroxychloride on tibia parameters of broilers ^1^.

Item	Mn as MnSO_4_ (mg/kg)	Mn as Manganese Hydroxychloride (mg/kg)						SEM	*p*-Value		
	100	0	20	40	60	80	100		ANOVA	Linear	Quadratic
Day 21											
Weight (g)	6.81	6.33	6.35	6.54	6.60	6.56	6.55	0.231	0.806	0.369	0.628
Length (mm)	76.07 ^a^	71.96 ^c^	72.35 ^bc^	74.43 ^ab^	75.29 ^a^	76.12 ^a^	75.17 ^a^	0.754	0.001	<0.001	0.172
Diameter (mm)	6.08	5.76	5.85	6.14	6.27	6.21	6.18	0.172	0.297	0.141	0.235
Strength (N)	132.72	124.19	123.30	129.78	128.11	130.02	132.31	2.865	0.159	0.025	0.932
Density index (g/cm^2^)	0.21 ^a^	0.19 ^b^	0.19 ^b^	0.20 ^ab^	0.20 ^ab^	0.20 ^ab^	0.21 ^a^	0.005	0.035	0.006	0.965
Day 42											
Weight (g)	19.37	18.82	18.40	18.40	17.86	19.68	18.19	0.609	0.358	0.975	0.739
Length (mm)	111.40	109.25	109.16	108.25	108.89	111.21	108.61	0.976	0.169	0.635	0.961
Diameter (mm)	9.59	10.37	9.08	9.54	9.19	9.87	9.66	0.417	0.398	0.678	0.136
Strength (N)	316.65	295.13	295.77	301.15	307.36	309.22	317.97	7.460	0.181	0.017	0.706
Density index (g/cm^2^)	0.30	0.29	0.28	0.29	0.29	0.29	0.29	0.005	0.807	0.829	0.769

^1^ Values represent the means of six pens (*n* = 6) per treatment. ^a, b, c^ Means within a row with different superscripts are significantly different at the *p*-value indicated for ANOVA.

**Table 8:** Effect of dietary supplementation with manganese hydroxychloride on Mn content in tissue of broilers on day 42 (mg/kg, dry matter basis) ^1^.

Item	Mn as MnSO_4_ (mg/kg)	Mn as Manganese Hydroxychloride (mg/kg)						SEM	*p*-Value		
	100	0	20	40	60	80	100		ANOVA	Linear	Quadratic
Serum	4.30	4.25	5.40	5.16	5.11	5.82	5.24	0.511	0.305	0.293	0.185
Heart	1.06 ^a^	0.64 ^b^	0.71 ^b^	0.70 ^b^	0.77 ^b^	0.79 ^b^	1.05 ^a^	0.065	<0.001	<0.001	0.131
Liver	10.40 ^ab^	9.16 ^b^	9.43 ^b^	9.59 ^b^	10.55 ^ab^	11.60 ^a^	11.66 ^a^	0.577	0.015	0.001	0.668
Breast muscle	0.42	0.37	0.38	0.40	0.41	0.41	0.39	0.023	0.703	0.371	0.205
Kidney	7.80 ^a^	5.68 ^d^	6.36 ^c^	6.76 ^bc^	6.80 ^bc^	6.83 ^bc^	7.24 ^ab^	0.230	<0.001	<0.001	0.008
Tibia	10.22 ^a^	4.74 ^c^	5.75 ^c^	5.90 ^c^	8.24 ^b^	8.36 ^ab^	8.39 ^ab^	0.619	<0.001	<0.001	0.375

^1^ Values represent the means of six pens (*n* = 6) per treatment. ^a–d^ Means within a row with different superscripts are significantly different at the *p*-value indicated for ANOVA.

## Data Availability

The data presented in this study are available on request from the corresponding author.
